# Role of *Lactiplantibacillus plantarum* UBLP-40, *Lactobacillus rhamnosus* UBLR-58 and *Bifidobacterium longum* UBBL-64 in the Wound Healing Process of the Excisional Skin

**DOI:** 10.3390/nu15081822

**Published:** 2023-04-10

**Authors:** Dimitrios Panagiotou, Eirini Filidou, Maria Gaitanidou, Gesthimani Tarapatzi, Michail Spathakis, Leonidas Kandilogiannakis, George Stavrou, Konstantinos Arvanitidis, Joulia K. Tsetis, Persefoni Gionga, Anne D. Shrewsbury, Vangelis G. Manolopoulos, Dora Kapoukranidou, Konstantinos Lasithiotakis, George Kolios, Katerina Kotzampassi

**Affiliations:** 1 Department of Surgery, School of Medicine, University of Crete, 71003 Heraklion, Greece; dimpanagiotou@yahoo.gr (D.P.); k.lasithiotakis@uoc.gr (K.L.); 2 Laboratory of Pharmacology, Faculty of Medicine, Democritus University of Thrace, 68100 Alexandroupolis, Greece; efilidou@hotmail.com (E.F.); mariegaitanidou@gmail.com (M.G.); mtarapagi@gmail.com (G.T.); michael.spathakis@outlook.com (M.S.); lkandilo@med.duth.gr (L.K.); karvanit@med.duth.gr (K.A.); emanolop@med.duth.gr (V.G.M.); gkolios@med.duth.gr (G.K.); 3 Individualised Medicine & Pharmacological Research Solutions Center (IMPReS), 68100 Alexandroupolis, Greece; 4 Department of Surgery, Aristotle University of Thessaloniki, 54636 Thessaloniki, Greece; stavgd@gmail.com (G.S.); gionga.persa@gmail.com (P.G.); a_shrewsbury@yahoo.com (A.D.S.); 5 Pharmacist MSc, Uni-Pharma S.A., 14564 Athens, Greece; jtsetis@uni-pharma.gr; 6 Department of Physiology, Faculty of Medicine, Aristotle University of Thessaloniki, 54124 Thessaloniki, Greece; dkapoukr@gmail.com

**Keywords:** probiotics, skin trauma, wound healing, microbiota, inflammation, angiogenesis, *Lactiplantibacillus plantarum* UBLP-40, *Lactobacillus rhamnosus* UBLR-58, *Bifidobacterium longum* UBBL-64

## Abstract

The probiotics *Lactiplantibacillus plantarum* UBLP-40, *Lactobacillus rhamnosus* UBLR-58 and *Bifidobacterium longum* UBBL-64 seem to promote wound healing when applied topically. Our aim was to investigate their effect on the mRNA expression of pro-inflammatory, healing and angiogenetic factors during the healing process of a standardized excisional wound model in rats. Rats subjected to six dorsal skin wounds were allocated to Control; *L. plantarum*; combined formula of *L. rhamnosus* plus *B. longum*; *L. rhamnosus*; and *B. longum* treatments, applied every two days, along with tissue collection. The pro-inflammatory, wound-healing, and angiogenetic factors of mRNA expression were assessed by qRT-PCR. We found that *L. plantarum* exerts a strong anti-inflammatory effect in relation to *L. rhamnosus*–*B. longum*, given alone or in combination; the combined regime of *L. rhamnosus*–*B. longum*, works better, greatly promoting the expression of healing and angiogenic factors than *L. plantarum*. When separately tested, *L. rhamnosus* was found to work better than *B. longum* in promoting the expression of healing factors, while *B. longum* seems stronger than *L. rhamnosus* in the expression of angiogenic factors. We, therefore, suggest that an ideal probiotic treatment should definitively contain more than one probiotic strain to speed up all three healing phases.

## 1. Introduction

It is increasingly acknowledged that skin wounds exert a direct effect on the local microbiota, leading to its disruption and reduction in its diversity, and thus the topical application of probiotics to restore the skin microbiota could be a promising and interesting approach. During recent years both in vivo and in vitro studies, as well as many experimental and a few clinical studies targeting the microbiome and its restoration, have supported a general consensus that the topical appliance of probiotics on cutaneous wounds of all kinds positively stimulates the wound healing process [1,2,3,4,5,6].

However, as there is heterogeneity among studies, many questions still remain unanswered regarding the type and dose of the probiotic used, especially at a time when the pharmaceutical industry is urgently looking for the most appropriate combination regime to provide the optimal healing effect by modulating the various stages of healing [3,7,8].

In a recently published experimental investigation performed by our study group, we evaluated the phase-by-phase wound healing properties of two different probiotic regimes applied topically: *Lactiplantibacillus plantarum* UBLP-40, and a commercially available formula of *Lactobacillus rhamnosus* UBLR-58 and *Bifidobacterium longum* UBBL-64 [9]. More specifically, we calculated the 3-D configuration of a standardized excisional wound in male Wistar rats throughout the wound healing process versus control, in an effort to compare the effectiveness of each probiotic regime, in each phase of healing. Our findings were a surprise: *L. plantarum* was found to play a significant role in the early inflammatory stage of healing, enabling re-epithelization to begin earlier in relation to the duo-probiotic regime. However, their roles reversed as the experiments progressed, with the latter duo-probiotic regime speeding up the cell migration and differentiation process for tissue reconstruction and, eventually, on Day 16, achieving a greater healing rate in comparison to *L. plantarum* [9].

Based on these initial findings, we decided to expand our investigation by designing a new set of experiments in order to further explore the influence of each of the three probiotics, i.e., *Lactiplantibacillus plantarum* UBLP-40, *Lactobacillus rhamnosus* UBLR-58 and *Bifidobacterium longum* UBBL-64 on the underlying mechanisms—at mRNA expression level—involved in the phase-by-phase wound healing process. In other words, we matched each probiotic with at least one mechanism/phase of healing in which it shows greater efficacy in speeding up healing.

## 2. Materials and Methods

### 2.1. Probiotic Treatment

The probiotics used in the present study were: *Lactiplantibacillus plantarum* UBLP-40, *Lactobacillus rhamnosus* UBLR-58, and *Bifidobacterium longum* UBBL-64. They were delivered as a fresh purified stock culture in the form of a dry powder containing 10^11^ cfu/gr, donated by the Pharmaceutical Company UniPharma, SA, Athens, Greece. For its use in the present experiment, exactly as in the previous one [9], a volume of 0.3 mL of normal saline 0.9% was freshly added to each pre-weighed gram of dry probiotic culture, making it a type of “ointment”, ready for application to the wound.

### 2.2. Probiotics Viability, Identity and Purity Testing

In order to verify the viability of the probiotic strains to be used, trypan-blue staining [10] was performed, and in order to verify their identity and purity, Gram staining was used as previously described [11].

### 2.3. Animals

Healthy male Wistar rats weighing 200–250 g were housed individually in polypropylene cages under controlled light, temperature and humidity conditions; a 12-h light–dark cycle at 22 ± 2 °C and 50 ± 2% humidity. Water and a standard laboratory chow diet were provided ad libitum, and the rats were allowed to acclimatize for a week. Housing, anesthesia, wound induction and post-operative care complied with the European Guidelines for the Care and Use of Laboratory Animals. The experimental protocol was approved by the Local Governmental Committee for the Control and Supervision of Experiments on Animals [EU Directive 2010/63/EU, Protocol registration number 227933(934)/06.05.2021].

### 2.4. Wound Induction

After intraperitoneal anesthesia [ketamine,100 mg/kg plus xylazine,10 mg/kg body weight], six full-thickness open excision wounds extending through the panniculus carnosus were surgically performed in the dorsal skin on each side of the midline, using a sterile 8 mm diameter biopsy punch, as previously described in detail [9]. A blinded operator applied either 0.3 mL of normal saline 0.9% or freshly prepared probiotic ointment on the excisional wound area, according to the allocation group [10^11^ cfu/gr per rat] and covered the wounds with a sterile gauze dressing reinforced with a self-adhesive bandage. Treatment was repeated every two days, the rats being under ether anesthesia, as previously described [9].

### 2.5. Study Design

1st experiment: Fifty-two rats, by means of random numbers, were allocated into: baseline (4 rats); placebo–control group (Ctrl-16 rats); single probiotic-treatment (*L. plantarum* group—16 rats); and duo-probiotics (*L. rhamnosus* and *B. longum*] group—16 rats).

2nd experiment: Sixty-four rats were allocated into: placebo–control group (Ctrl—16 rats); *L. plantarum* group—16 rats; *L. rhamnosus* group—16 rats; and *B. longum* group—16 rats.

The four baseline-group rats, upon wound induction, were immediately sacrificed with an anesthesia formula overdosing, being thus used as Day 0 for all groups. The wounded tissue was then en-bloc—full-thickness skin—excised using the same diameter [8 mm] biopsy punch and immediately snap-frozen for further processing. The same practice was followed on Days 2, 4, 8, and 16 on four rats from each group.

### 2.6. RNA Studies

#### 2.6.1. Total RNA Extraction and Purification

A small section of tissue, approximately 30–50 mg, was mechanically homogenized in 500 μL Nucleozol (MACHEREY-NAGEL, Düren, Germany). Total RNA from each sample was extracted according to the manufacturer’s instructions and as previously described [12]. In short, 200 μL of H_2_O was added to each sample, following incubation and a 15 min centrifugation at 12,000× *g*. Precipitation of total RNA was performed by adding 500 μL of isopropanol (Sigma-Aldrich, St. Louis, MO, USA) to each sample and centrifuging for 10 min at 12,000× *g*, and total RNA was washed twice with 75% ethanol. Total isolated RNA was diluted in RNase-free H_2_O. RNA concentration and purity were measured using a Q500 UV-Vis spectrophotometer (Quawell, San Jose, CA, USA). Potential DNA contaminations were eliminated using Deoxyribonuclease I (Recombinant Dnase I (RNase Free); TaKaRa, Kusatsu, Shiga, Japan) for 15 min. Inactivation of DNAse was accomplished by EDTA and heat treatment.

#### 2.6.2. cDNA Synthesis and Real-Time Reverse Transcription Polymerase Chain Reaction

cDNA synthesis was performed with the use of the PrimeScript RT Reagent Kit (Perfect Real Time) (TaKaRa, Kusatsu, Shiga, Japan) according to the manufacturer’s instructions. Briefly, 250 ng of total RNA was mixed with the 5× PrimeScript Buffer, reverse transcriptase, oligo dT primers, random hexamers, and RNase-free H_2_O and was then incubated at 37 °C for 15 min. Quantitative RT-PCR was performed for TNF-α, IL-17, IL-6, IL-10, IL-1b, TGF-β, COL-I, COL-III, α-SMA, CTGF, VEGF, EGFRF, EGF, PDGF to determine the mRNA expression gene-specifically. Table 1 shows the forward and reverse sequences of the oligonucleotide primers. Amplification occurred in a SaCycler-96 real-Time PCR system (Sacace Biotechnologies, Como, Italy) in a two-step cycling protocol with an annealing temperature of 60 °C. The normalization of the results was conducted using the 2^−ΔΔCt^ method, considering GAPDH as a reference gene.

### 2.7. Statistical Analysis

The normality of the data was verified by the Shapiro–Wilk test. One-way analysis of variance [ANOVA] was used for between and within-group comparisons using the Kruskal–Wallis test. Data are presented as mean ± standard error of the mean. For statistical analysis purposes, the GraphPad Prism software version 9.5.1 (GraphPad Software, San Diego, CA, USA, www.graphpad.com accessed on 20 February 2023) was used, while a *p*-value of less than 0.05 was considered statistically significant.

## 3. Results

### 3.1. 1st Experiment

#### 3.1.1. *L. plantarum* Reduces Inflammation More Efficiently than the Combo *L. rhamnosus*–*B. longum*

**Figure 1:**
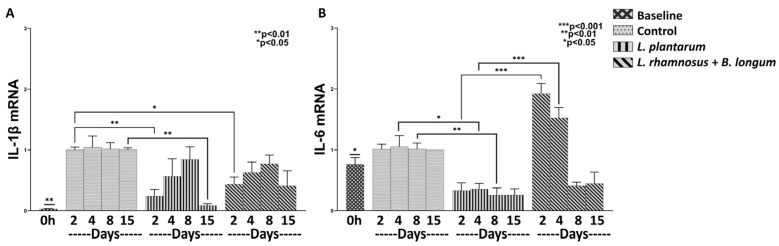
The effects of *L. plantarum* and the combination of *L. rhamnosus*–*B. longum* on the pro-inflammatory response. The mRNA expression of IL-1β (**A**) and IL-6 (**B**) during the course of the healing process. Results are presented as means with SEM. IL-1 β Baseline: ** *p* < 0.01 compared to Control (Days 2–15), *L. plantarum* (Days 2–8) and *L. rhamnosus* and *B. longum* (Days 2–15); IL-6 Baseline: * *p* < 0.05 compared to *L. plantarum* (Days 2–8) and *L. rhamnosus* and *B. longum* (Days 2–8).

In order to clarify whether *L. plantarum* alone or the combined formula *L. rhamnosus*–*B. longum* can affect the inflammatory response, we examined the mRNA expression of the pro-inflammatory cytokines TNF-α, IL-1β, IL-6, IL-10 and IL-17 in relation to control. Regarding the baseline (time 0) mRNA expression of IL-1β (Figure 1A), it was found statistically significantly downregulated, compared to Control (Days 2–15; *p* < 0.01), to *L. plantarum* (Days 2–8; *p* < 0.01) and to combo regime *L. rhamnosus*–*B. longum* (Days 2–15; *p* < 0.01). As for the IL-6 baseline mRNA expression (Figure 1B), it was found statistically significantly upregulated in relation to *L. plantarum* (Days 2–8; *p* < 0.05) and to *L. rhamnosus* –*B. longum* on Day 8 of treatment (*p* < 0.05). However, the IL-6 baseline mRNA expression was also observed to be statistically significantly downregulated in relation only to *L. rhamnosus*–*B. longum*, on Days 2 and 4 of treatment (*p* < 0.05).

Regarding the efficacy of each probiotic regime on the magnitude of the inflammatory response, it was found to generally depend on the specific probiotic used and the day of the healing process. More specifically, *L. plantarum* exhibited an anti-inflammatory effect, as it was found to reduce the mRNA expression of IL-1β and IL-6, with more profound results by the end of the experiment. Specifically, it was observed that *L. plantarum* statistically significantly downregulated the IL-1β mRNA expression by Day 15 (0.08-fold, ±0.03, *p* < 0.01; Figure 1A), and the IL-6 mRNA expression by Day 8 (0.25-fold, ±0.11, *p* < 0.01; Figure 1B), when compared to control. Similarly, the probiotic mixture of *L. rhamnosus*–*B. longum* exerted a similar decrease on the IL-1β mRNA expression (Day 2: 0.43-fold, ±0.11, *p* < 0.05; Figure 1A) when compared to control. On the other hand, the combo regime led to an increase in the IL-6 mRNA when compared to *L. plantarum*, mainly during the inflammatory phase of the healing process (Day 2: 1.92-fold, ±0.16, *p* < 0.001; Day 4: 1.52-fold, ±0.17, *p* < 0.001; Figure 1B). These findings clearly suggest a beneficial effect of *L. plantarum* during the early inflammatory phase in relation to the combo regime.

Finally, the mRNAs of the inflammatory factors TNF-α, IL-17 and IL-10 were not expressed, either by the control group or by the probiotic-treated wounds.

#### 3.1.2. The Combo Regime Works Better than *L. plantarum* in Promoting the Expression of Healing Factors

**Figure 2:**
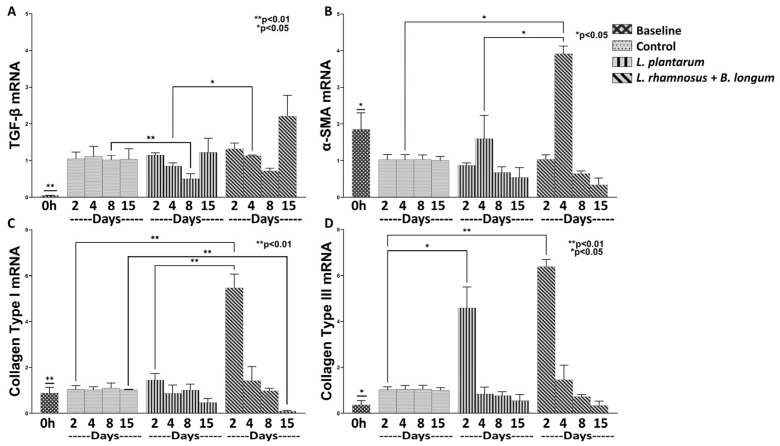
The effects of *L. plantarum* and the combination of *L. rhamnosus*–*B. longum* on the wound healing response. The mRNA expression of TGF-β (**A**), α-SMA (**B**), Collagen Type I (**C**) and Collagen Type III (**D**) during the healing process. Results are presented as means with SEM. TGF-β Baseline: ** *p* < 0.01 compared to Control (Days 2–15), *L. plantarum* (Days 2–8) and *L. rhamnosus* and *B. longum* (Days 2–15); α-SMA Baseline: * *p* < 0.05 compared to *L. plantarum* (Days 2, 8 and 15) and *L. rhamnosus* and *B. longum* (Days 4–8); Collagen Type I Baseline: ** *p* < 0.01 compared to *L. rhamnosus* and *B. longum* (Days 2 and 15); Collagen Type III Baseline: * *p* < 0.05 compared to *L. plantarum* (Day 2), and *L. rhamnosus* and *B. longum* (Day 2).

We then investigated the efficacy of the aforementioned probiotics on wound healing. Both regimes were found to significantly promote the healing process by means of regulating the mRNA expression of TGF-β, α-SMA, Collagen Type I and Type III (Figure 2).

Regarding the baseline values of TGF-β, Collagen Type I and Type III mRNA expression (Figure 2A,C,D), they were found to be statistically significantly reduced in relation to all treatments and study phases. On the contrary, α-SMA baseline mRNA expression (Figure 2B) was found statistically significantly higher compared to *L. plantarum* values on Days 2, 8 and 15 (*p* < 0.05) and to *L. rhamnosus*–*B. longum* on Day 8 (*p* < 0.05).

As far as the effect of each probiotic regime is concerned throughout the healing process, it was apparent that they significantly promoted the initiation of the healing process, while later, they contributed to the attenuation of these factors, possibly reducing hypertrophic wound scar formation. Specifically, when compared to control, *L. plantarum* resulted in a mild, but statistically significant, induction of Collagen Type III mRNA expression on Day 2 (4.59-fold, ±0.90, *p* < 0.05; Figure 2D) while also reducing the TGF-β expression on Day 8 (0.50-fold, ±0.13, *p* < 0.01; Figure 2A). On the other hand, although we observed no statistically significant difference in the expression of TGF-β on Day 4 between the control and the combo regime, the latter significantly enhanced its expression when compared to *L. plantarum* (1.12-fold, ±0.02, *p* < 0.05; Figure 2A). Regarding the rest of the studied healing factors, the combo regime treatment resulted in a statically significant expression of α-SMA on Day 4 (3.91-fold, ±0.20, *p* < 0.05; Figure 2B) and of Collagen Type I on Day 2 (5.46-fold, ±0.60, *p* < 0.01; Figure 2C) in relation to both control and *L. plantarum*. Finally, although we observed that the combo regime statistically significantly upregulated the Collagen Type III on Day 2 (6.38-fold, ±0.31, *p* < 0.01; Figure 2D) in relation to the control, there was no statistically significant difference between the effects of *L. plantarum* and the combo regime. In this respect, we concluded that the combo probiotics treatment was more effective in promoting both the healing process and the later resolution of scar formation.

#### 3.1.3. The Combo Regime *L. rhamnosus*–*B. longum*, but Not the *L. plantarum* Strongly Enhance the Angiogenesis Process

**Figure 3:**
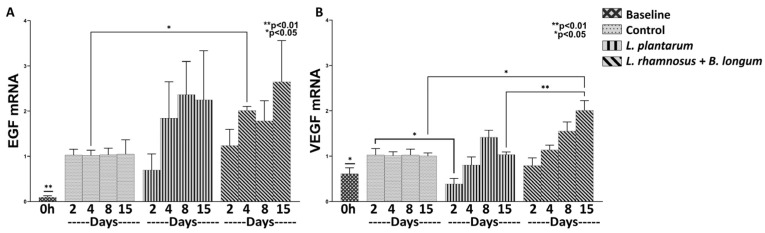
The effects of *L. plantarum* and the combination of *L. rhamnosus* and *B. longum* on angiogenesis. The mRNA expression of EGF (**A**) and VEGF (**B**) during the healing process. Results are presented as means with SEM. EGF Baseline: ** *p* < 0.01 compared to Control (Days 2–15), *L. plantarum* (Days 2–15) and *L. rhamnosus*–*B. longum* (Days 2–15); VEGF Baseline: * *p* < 0.05 compared to *L. plantarum* (Days 2 and 8) and *L. rhamnosus*–*B. longum* (Days 4–15).

Angiogenesis is initiated immediately after tissue injury and is mediated throughout the wound-healing process; thus, we further analyzed the efficacy of *L. plantarum* and the combo regime of *L. rhamnosus*–*B. longum* on the mRNA expression of the angiogenetic factors CTGF, VEGF, EGFRF, EGF, and PDGF. A significant upregulation of EGF mRNA (Control, Days 2–15: *p* < 0.01; *L. plantarum*, Days 2–15: *p* < 0.01; Combo Regime, Days 2–15: *p* < 0.01), and to a lesser extent of VEGF mRNA (*L. plantarum*, Days 2, 8: *p* < 0.05; Combo Regime, Days 4–15: *p* < 0.05) was clear in relation to baseline values (Figure 3); the CTGF, EGFRF, and PDGF factors were not expressed at all.

*L. plantarum* treatment appeared to be non-active in relation to control treatment on the expression of EGF and VEGF (Figure 3A,B). Specifically, although it had no effect on the expression of EGF (Figure 3A), it did downregulate the mRNA expression of VEGF on Day 2 when compared to the control (0.38-fold, ±0.12, *p* < 0.05; Figure 3B). Quite the opposite, the combo probiotic formulation led to a steady increase, over time, in mRNA expression of both EGF on Day 4 (2.01-fold, ±0.09, *p* < 0.05; Figure 3A) in relation to control and VEGF on Day 15 (2.64-fold, ±0.91; Figure 3B) when compared to control (*p* < 0.05) and the *L. plantarum* treatment (*p* < 0.01).

### 3.2. 2nd Experiment

#### 3.2.1. *L. plantarum* Exerts a Strong Anti-Inflammatory Effect Compared to Both *L. rhamnosus* and *B. longum*

**Figure 4:**
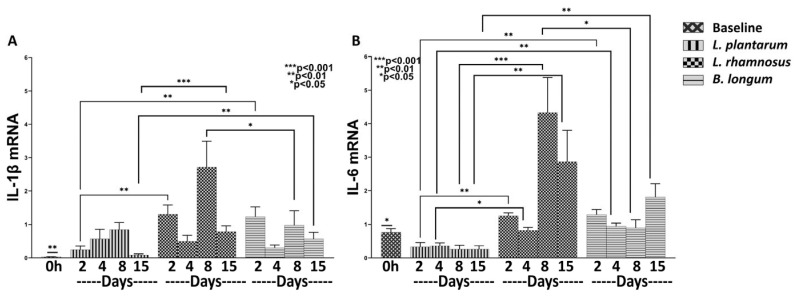
The effects of *L. plantarum*, *L. rhamnosus* and *B. longum* on the pro-inflammatory response. The mRNA expression of IL-1β (**A**) and IL-6 (**B**) during the healing process. Results are presented as means with SEM. IL-1β Baseline: ** *p* < 0.01 compared to *L. plantarum* (Days 2–8), *L. rhamnosus* (Days 2–15) and *B. longum* (Days 2–15); IL-6 Baseline: * *p* < 0.05 compared to *L. plantarum* (Days 2–15), *L. rhamnosus* (Days 8 and 15) and *B. longum* (Day 15).

Having shown that *L. plantarum* has a better anti-inflammatory effect in relation to the combo regime *L. rhamnosus*–*B. longum*, we proceeded to further assess the role of each separately in relation to *L. plantarum*. The expression of IL-1β mRNA (*L. plantarum* Days 2–8: *p* < 0.01; *L. rhamnosus* Days 2–15: *p* < 0.01; *B. longum* Days 2–15: *p* < 0.01), as well as of IL-6 mRNA (*L. plantarum* Days 2–15: *p*< 0.05; *L. rhamnosus* Days 8, 15: *p* < 0.05; *B. longum* Day 15: *p* < 0.05) was found upregulated compared to the baseline values (Figure 4).

As far as the effects of each probiotic strain are concerned, our results indicate that *L. rhamnosus,* as well as *B. longum,* increased the overall mRNA expression of IL-1β and IL-6 in relation to *L. plantarum*. Specifically, *L. rhamnosus* statistically significantly increased the expression of IL-1β on Days 2 and 15 (Day 2: 1.30-fold, ±0.27, *p* < 0.01 and Day 15: 0.78-fold, ±0.18, *p* < 0.001; Figure 4A) and of IL-6 during all days (Day 2: 1.25-fold, ±0.09, *p* < 0.01; Day 4: 0.81-fold, ±0.09, *p* < 0.05; Day 8: 4.32-fold, ±0.23, *p* < 0.05; Day 15: 2.86-fold, ±0.93, *p* < 0.01; Figure 4B), compared to *L. plantarum*. Regarding *B. longum*, it statistically significantly increased the mRNA expression of IL-1β on Days 2 and 15 (Day 2: 1.23-fold, ±0.29, *p* < 0.01; and Day 15: 0.56-fold, ±0.19, *p* < 0.01; Figure 4A) and of IL-6 on Days 2, 4 and 15 (Day 2: 1.29-fold, ±0.15, *p* < 0.01; Day 4: 0.94-fold, ±0.09, *p* < 0.01; Day 15: 1.81-fold, ±0.39, *p* < 0.01; Figure 4B) in relation to *L. plantarum*. In other words, *L. plantarum* appears to exert by far the greatest anti-inflammatory action, followed by *B. longum*.

#### 3.2.2. *L. rhamnosus* Works Better than *B. longum* in Promoting Expression of Healing Factors

**Figure 5:**
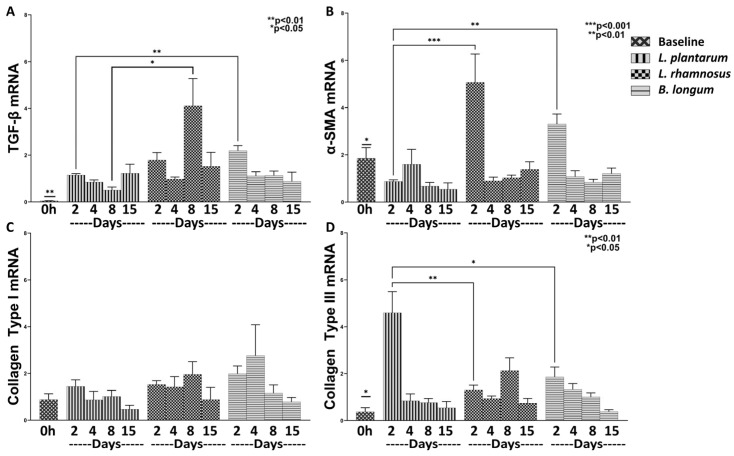
The effects of *L. plantarum*, *L. rhamnosus* and *B. longum* on the wound healing response. The mRNA expression of TGF-β (**A**), α-SMA (**B**), Collagen Type I (**C**) and Collagen Type III (**D**) during the healing process. Results are presented as means with SEM. TGF-β Baseline: ** *p* < 0.01 compared to *L. plantarum* (Days 2–15), *L. rhamnosus* (Days 2–15) and *B. longum* (Days 2–15); α-SMA Baseline: * *p* < 0.05 compared to *L. plantarum* (Days 2, 8 and 15), *L. rhamnosus* (Day 2) and *B. longum* (Day 2); Collagen Type III Baseline: * *p* < 0.05 compared to *L. plantarum* (Days 2–18), *L. rhamnosus* (Days 2–15), and *B. longum* (Days 2–8).

In the previous experiment, the combo regime of *L. rhamnosus*–*B. longum* exhibited better healing effects in relation to *L. plantarum*. Thus, we further investigated the role of each probiotic in the management of the healing process by measuring the TGF-β, α-SMA, Collagen Type I and Type III mRNA expression. We observed, as previously, an increase in the mRNAs of TGF-β (*L. plantarum* Days 2, 8, 15: *p* < 0.01; *L. rhamnosus* Days 2–15: *p* < 0.01; *B. longum* Days 2–15: *p* < 0.01), and of Collagen Type III (*L. plantarum* Days 2–8: *p* < 0.05; *L. rhamnosus* Days 2–15: *p* < 0.05; *B. longum* Days 2–8: *p* < 0.05), and a decrease of α-SMA (*L. plantarum* Day 2, 8, 15: *p* < 0.05; *L. rhamnosus* Day 2: *p* < 0.05; *B. longum* Day 2: *p* < 0.05) in relation to baseline measurements (Figure 5).

Regarding comparisons among the three probiotics: *L. rhamnosus* and much more *B. longum,* significantly increased the mRNA expression of TGF-β (*L. rhamnosus* Day 8: 4.10-fold, ±1.16, *p* < 0.05; *B. longum* Day 2: 2.19-fold, ±0.21, *p* < 0.01; Figure 5A) in relation to *L. plantarum*. In addition, *B. longum*, and much more *L. rhamnosus*, significantly increased the mRNA expression of α-SMA (*L. rhamnosus* Day 2: 5.06-fold, ±1.19, *p* < 0.01; *B. longum* Day 2: 3.30-fold, ±0.42, *p* < 0.05; Figure 5B), in comparison to *L. plantarum*. These findings support the hypothesis that both *L. rhamnosus* and *B. longum* exert a more active role in the progression of the wound healing process rather than in the inflammatory phase, each acting through the expression of a different healing factor. However, it should be noted that *L. rhamnosus* was found to significantly increase TGF-β on Day 8 in relation to *B. longum* (Figure 5A), thus raising questions for further research regarding the possibility of hypertrophic scarring induction.

Collagen Type I mRNA expression was similar, independently of the probiotic treatment applied (Figure 5C). However, *L. plantarum* induced a more profound upregulation of Collagen Type III mRNA expression compared to that induced by *L. rhamnosus* or *B. longum*. In addition, it is of interest to comment that, although the combo regime of *L. rhamnosus* –*B. longum* induced a significantly high expression of Collagen Type III compared to *L. plantarum* (Figure 2D), when applied separately, they both showed a significantly lower expression relative to *L. plantarum* (Figure 5D).

Collectively viewing our results, *L. rhamnosus* appears to have a more profound effect on the expression of healing factors TGF-β and α-SMA; there is, however, a small question about its ability to inhibit hypertrophic scarring formation.

#### 3.2.3. *B. longum* Seems Stronger in Promoting the Expression of Angiogenic Factors Compared to *L. rhamnosus*

The combo regime *L. rhamnosus*–*B. longum* was found to strongly enhance angiogenesis throughout the 15-day experiment. Therefore, we proceeded to further investigate the effects of the two components, *L. rhamnosus* and *B. longum,* separately on the mRNA expression of EGF and VEGF in relation to *L. plantarum*.

**Figure 6:**
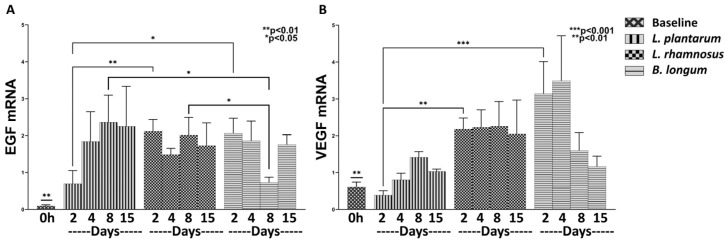
The effects of *L. plantarum*, *L. rhamnosus* and *B. longum* on angiogenesis. The mRNA expression of EGF (**A**) and VEGF (**B**) during the healing process. Results are presented as means with SEM. EGF Baseline: ** *p* < 0.01 compared to *L. plantarum* (Days 2–15), *L. rhamnosus* (Days 2–15) and *B. longum* (Days 2–15); VEGF Baseline: * *p* < 0.05 compared to *L. plantarum* (Day 8), *L. rhamnosus* (Days 2–15) and *B. longum* (Days 2–8).

Initially, we observed a significant upregulation of EGF and VEGF mRNA expression by *L. rhamnosus* and by *B. longum*, but not by *L. plantarum*, compared to Day 0 (EGF: *L. plantarum* Days 2–15: *p* < 0.01; *L. rhamnosus* Days 2–15: *p* < 0.01; *B. longum* Days 2–15: *p* < 0.01; and VEGF: *L. plantarum* Day 8: *p* < 0.05; *L. rhamnosus* Days 2–15: *p* < 0.05; and *B. longum* Days 2–8: *p* < 0.05; Figure 6).

Both *L. rhamnosus* and *B. longum* exhibited quite similar, significant upregulation of EGF mRNA expression on Day 2 (*L. rhamnosus*: 2.11-fold, ±0.31, *p* < 0.01; *B. longum*: 2.06-fold, ±0.40, *p* < 0.05; Figure 6A), with no further increase over time, in relation to *L. plantarum*, which reached the same high levels only after Day 4. Unexpectedly, *B. longum* significantly reduced the EGF mRNA expression on Day 8 (0.73-fold, ±0.14, *p* < 0.05; Figure 6A) compared to both *L. plantarum* and *L. rhamnosus*, only to return again to high levels on Day 15.

Regarding VEGF mRNA expression, *L. rhamnosus* presented a significant upregulation on Day 2, the values remaining steadily high throughout the 15-day experiment in relation to *L. plantarum* (*L. rhamnosus* Day 2: 2.18-fold, ±0.30, *p* < 0.01; Figure 6B). *B. longum* exhibited significantly higher values in relation to *L. plantarum* (*B. longum* Day 2: 3.14-fold, ±0.87, *p* < 0.001; Figure 6B) up to Day 4 and downregulated thereafter.

These findings lead us to suggest that *L. rhamnosus* and *B. longum* almost equally promote angiogenesis in relation to *L. plantarum*. However, the short-duration but higher-value VEGF mRNA expression induced by *B. longum* still needs further clarification.

## 4. Discussion

In the present study, we present the positive effects of *Lactiplantibacillus plantarum*, *Lactobacillus rhamnosus* and *Bifidobacterium longum*, as well as of the probiotic mixture *L. rhamnosus* and *B. longum* on the healing process of an excisional skin trauma in Wistar rats. Our results overall showed that these probiotic strains are able to enhance wound healing by regulating inflammatory responses, promoting angiogenesis and inducing the restoration of the tissue. It is worth mentioning that each strain had a unique effect on the factors studied, which changed over the course of the wound healing process, contributing to the activation of the different cell populations of the skin tissue. Specifically, we observed that although *L. plantarum* was more effective in downregulating the pro-inflammatory IL-1β and ΙL-6, the combination of *L. rhamnosus* and *B. longum* had a stronger induction of healing factors—α-SMA, Collagen Type I and III—as well as the angiogenetic VEGF, in relation to *L. plantarum*. In addition, we also observed that the effect of the probiotic combination in promoting healing could probably be attributed to the presence of *L. rhamnosus*, while the favorable angiogenetic effect could be attributed to the presence of *B. longum*. It should also be noted that our mRNA results are in agreement with the observed macroscopic healing (Supplementary Figure S1), showing that the combo regime and the *L. rhamnosus* or *B. longum* alone greatly enhance the healing process.

It is widely known that the skin microbiota, aside from attributing to metabolic pathways and protecting against pathogens, can also promote local homeostasis by influencing immune responses [13]. Our team has already published the benefits of topically applying these probiotics to the wound site, both regarding the healing rate and the risk of infection [9]. Here, we further proceeded to analyze the interplay of *L. plantarum* or *L. rhamnosus* and/or *B. longum* with the skin cell populations and downward immunological cascades. More specifically, skin wound healing requires the coordination of several cell types at precise stages comprising four phases: coagulation, inflammation, proliferation, and remodeling [14]. Microbiota regulates skin homeostasis by influencing a variety of cell signaling and homeostatic processes, including keratinocyte proliferation, epithelial differentiation, and epidermal blood vessel development [15]. Topical application of probiotics in laboratory animals seems to significantly improve the healing process, and each strain works differently and more effectively in different phases of healing [16,17,18]. This observation was also confirmed by the present study, as we showed that *L. plantarum* primarily affects the inflammatory phase of wound healing by reducing the expression of pro-inflammatory cytokines, while *L. rhamnosus* and *B. longum* mainly promote the healing and the angiogenesis phases, as they both greatly induce the expression of several healing and angiogenic factors.

The wound healing process begins with hemostasis—included in the inflammatory stage—which comprises a wide range of regulating factors and cytokines [19,20,21]. We previously showed that *L. plantarum* plays an important role in the early inflammatory stage of healing, allowing faster epithelialization, resulting in the shrinkage of the injured area and protection against possible pathogen entry [9]. Furthermore, it is shown that this strain is involved in the alertness of the immune system and healing process without causing serious inflammatory cascades [11]. The present results strongly support previous findings that the specific anti-inflammatory action of *L. plantarum* may originate from the downregulation of pro-inflammatory cytokines IL-6 and IL-1β without significantly affecting the anti-inflammatory IL-10 or the pro-inflammatory TNF-α. Indeed, our results are in agreement with a previous study showing that *L. plantarum* improves diabetic wound healing through the regulation of immune cells and pro-inflammatory cytokine production [22]. In addition, we also showed that *L. plantarum* had a superior effect on reducing inflammation when compared to *L. rhamnosus* and/or *B. longum*, suggesting that this probiotic might have a pivotal role in regulating the host’s immunological responses.

During the wound healing process, the promotion of angiogenesis and restoration of trauma are necessary and include the proliferation and migration of keratinocytes, the proliferation of fibroblasts, matrix deposition, and angiogenesis [23,24,25,26]. Fibroblasts further stimulate keratinocyte proliferation and migration through KGF, EGF, and fibronectin, a process enhanced by the presence of VEGF and FGF-β [27,28,29], while a wide variety of other cells, such as neutrophils and macrophages, contribute by releasing CXCL2, CXCL3, PDGF, VEGF, TGF-α, TGF-β, TNF-α, and interleukins IL-1β, IL-6, IL-8 and IL-10 [29,30,31].

Regarding the effect of the studied probiotics on angiogenesis, we observed that the combo regime of *L. rhamnosus*–*B. longum* was more effective in promoting EGF, VEGF and their downstream angiogenetic mechanisms on skin trauma healing compared to *L. plantarum*. Nonetheless, the available data on angiogenesis are sparse. A previous study reported that topical administration of *L. plantarum* on diabetic foot ulcers promoted more efficient wound healing and angiogenesis as microvessel density was improved [32]. Regarding *L. rhamnosus*, its topical administration on a mouse model of skin wound healing resulted in increased wound healing rates, along with enhanced angiogenesis, as VEGF was found to be upregulated and blood flow was significantly increased [33]. Lam et al. reported similar results on gastric ulcers, where treatment with *L. rhamnosus* led to increased formation of microvessels [34]. The impact of *B. longum* monotherapy or combination on angiogenesis has not yet been fully elucidated.

The next stage of the healing process involves the remodeling of ECM [35], resulting in scar formation and restoration of the barrier. This phase is characterized by the replacement of type III by type I collagen [36] and a reduction in cell density caused by apoptotic mechanisms [37]. In addition, both increased metabolic activity is observed, and contraction of individual capillaries into larger vessels, which is controlled by multiple cell types that secrete numerous growth factors, cytokines, and chemokines to achieve closure and functional restoration of the barrier [38]. We previously showed that the *L. rhamnosus* and *B. longum* combination accelerated the process of cell migration and differentiation for tissue reconstruction and finally, on Day 16, achieved the highest percentage of healing [9]. Generally, studies related to microbial symbiont mixtures containing both lactic acid bacteria and yeasts in experimental animals show enhanced wound healing as collagen levels are increased and tissue “reconstruction” occurs faster [3,39,40,41,42].

The present study does indeed show that the mRNA of α-SMA, Collagen Type I and Type III were initially upregulated by the probiotics during the first days of the trauma, probably in order to promote the healing process, and then significantly reduced over time, contributing to the wound healing. In addition, we also observed that the expression levels of TGF-β were regulated differently by each probiotic strain throughout the healing process, highlighting once more their strain-specific properties. However, in most of the studied factors, the effect of *L. rhamnosus* was stronger at Day 8, suggesting an important yet delayed contribution to the wound healing process, which was even further enhanced when *B. longum* was co-administrated, as significant healing results were observed as early as Day 2. Specifically, we observed that the combination of *L. rhamnosus* and *B. longum* had a greater effect in inducing the expression of TGF-β, α-SMA, Collagen Type I and Type III than *L. plantarum*, suggesting that these two probiotics have a more crucial role in tissue remodeling, and out of the two, we consider that the better healing outcome could possibly be attributed to the presence of *L. rhamnosus*. Our results are in agreement with previous studies showing that combinational probiotic treatments greatly improve the healing processes [9,43,44,45].

We once again provide evidence that different probiotic strains can have superior effects on every stage of the wound reconstruction process by excreting stronger signals and being implicated in more intercellular interactions. Although it is known that mixtures may be more beneficial as the properties of different microorganisms are combined and probiotic performance is increased [11,46], the key interactions between the human host and microflora, as well as the most suitable probiotic combination, based on the effects of each strain in the wound healing phases, have yet to be elucidated.

Certainly, it would be of great interest to combine the mRNA findings with those of immune-histochemistry, such as staining for M1 macrophages for the assessment of inflammatory reaction, for blood vessels density to assess angiogenesis and for collagen, α-SMA and other stromal markers for the assessment of tissue remodeling. This is a certain limitation of our study, and future research is needed.

## 5. Conclusions

In the present study, we presented the effects of the probiotics *L. plantarum*, *L. rhamnosus* and *L. longum* on the implicated molecular pathways during the phases of skin wound healing.

We conclude that each probiotic exerts its benefits through different modes of action and in different phases of healing: *L. plantarum* is more efficient during the early inflammation phase than *L. rhamnosus* and/or *B. longum*, as it greatly downregulates IL-1β and IL-6 pro-inflammatory cytokines. The combined regime of *L. rhamnosus* and *B. longum* greatly promotes the healing and angiogenesis phases in relation to *L. plantarum*, as it significantly increases the expression of several ECM remodeling and angiogenetic factors; when given separately, *L. rhamnosus* exerts a stronger effect on the healing process and *B. longum* on the angiogenic process. In addition, our mRNA results are in agreement with the observed macroscopic healing, suggesting that probiotic treatments are able to indeed enhance the healing process and, therefore, could be of great benefit to the host.

We, therefore, suggest that an ideal probiotic treatment to modulate various healing phases should definitively contain a well-studied *L. plantarum* strain to speed up the inflammatory phase, an *L. rhamnosus* strain to promote mainly tissue healing and remodeling and a *B. longum* strain to promote mainly angiogenesis, although there is an overlap in their activities.

Further research is required both to elucidate in depth the specific healing mechanisms and to test the reproduction of results or produce better results in relation to different concentrations of probiotics. We are already working to this end.

## Figures and Tables

**Table 1:** Forward and Reverse sequences of primers used in q-PCR.

Gene	Forward Primer	Reverse Primer
TNF-α	TGGGCTCCCTCTCATCAGTT	CTTGGTGGTTTGCTACGACG
Gapdh	AGTGCCAGCCTCGTCTCATA	GGTAACCAGGCGTCCGATA
IL-1b	GCAATGGTCGGGACATAGTT	AGACCTGACTTGGCAGAGGA
Col-1	ATCAGCCCAAACCCCAAGGAGA	CGCAGGAAGGTCAGCTGGATAG
Col-3	TGATGGGATCCAATGAGGGAGA	GAGTCTCATGGCCTTGCGTGTTT
α-SMA	TGACCCAGATTATGTTTGAG	AGATAGGCACGTTGTGAGTC
TGF-β1	CATTTGGAGCCTGGACACACA	GCTTGCGACCCACGTAGTAGAC
CTGF	CAGCATGGACGTTCGTCTG	AACCACGGTT TGGTCCTTGG
EGFR	TACCTGAGAGACCGCCATA	TGCTTCTTCTGCTTCCCTA
VEGF	GCAATGATGAAGCCCTGGAGT	CTGAACAAGGCTCACAGTGATTTT
IL-6	GCCCTTCAGGAACAGCTATGA	TGTCAACAACATCAGTCCCAAGA
IL-17	ACTTTCCGGGTGGAGAAGAT	CTTAGGGGCTAGCCTCAGGT
IL-10	GGCCATTCCATCCGGGGTGA	AAGGCAGCCCTCAGCTCTCG
EGF	TGTGGGCTGAGAAGAAGCTG	GAGTACCAGATCTGCCGCT
PDGF	TTCTTGATCTGGCCCCCAT	TTGACGCTGCTGGTGTTACAG
IL-8	CTTTGTCCATTCCCACTTCTGA	TCCCTAACGGTTGCCTTTGTAT

## Data Availability

The data presented in this study are available on request from the corresponding author.

## Supplementary Materials

The following supporting information can be downloaded at: https://www.mdpi.com/article/10.3390/nu15081822/s1, Figure S1: Representative photos of the excisional wounds in the five groups on Day 0 [control], Day 4 [termination of inflammatory phase] and Day 16.
