# Supplementary figures and images for: Role of Lactiplantibacillus plantarum UBLP-40, Lactobacillus rhamnosus UBLR-58 and Bifidobacterium longum UBBL-64 in the Wound Healing Process of the Excisional Skin
Source: Nutrients. 2023 Apr 10;15(8):1822. doi: 10.3390/nu15081822 (PMC10141733; doi:10.3390/nu15081822)

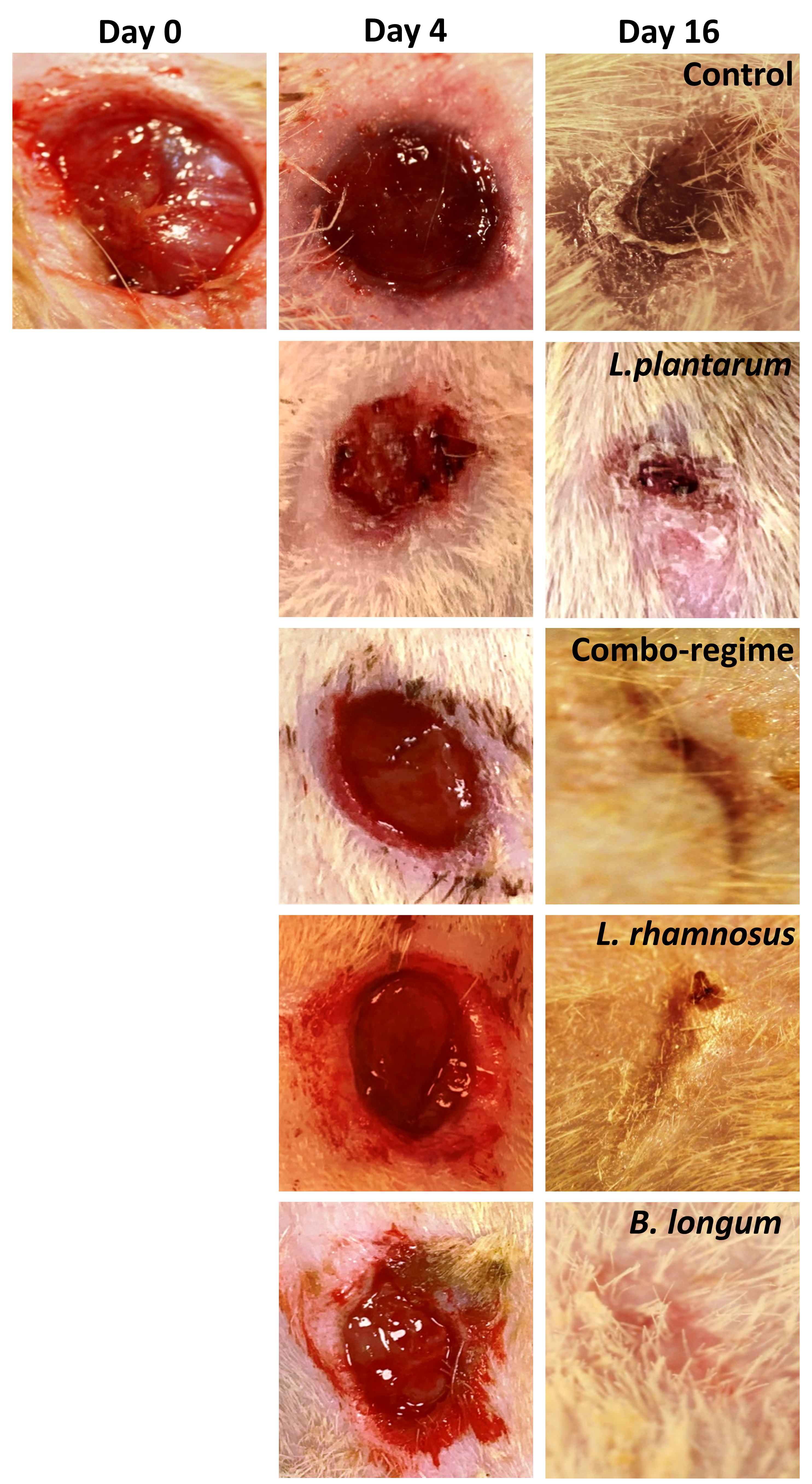

Supplement: Supplementary file 1 [file nutrients-15-01822-s001.zip › nutrients-2283614-supplementary/Supplementary Figure S1.tif]
